# Price dispersion of generic medications

**DOI:** 10.1371/journal.pone.0225280

**Published:** 2019-11-18

**Authors:** John J. J. Bernstein, Gerhard B. Holt, Joseph Bernstein

**Affiliations:** 1 Haverford High School, Haverford, Pennsylvania, United States of America; 2 Independent Scholar, Philadelphia, Pennsylvania, United States of America; 3 University of Pennsylvania, Philadelphia, Pennsylvania, United States of America; York University, CANADA

## Abstract

Generic pharmaceuticals should have very little price dispersion. Economics’ Law of One Price suggests that identical goods, in the absence of trade frictions and under conditions of free competition and price flexibility, should sell for the same price, and the FDA ensures that generics are identical. In this study, we examine whether generic pharmaceuticals indeed have the low price dispersion that theory predicts, and if not, whether the dispersion seen for pharmaceuticals used to treat neuropsychiatric conditions is substantially higher than that of other drugs. Such a difference may offer an explanation for the price dispersion seen: namely, a strategy that takes advantage of buyers’ cognitive constraints and impaired ability to comparison shop. We thus assembled a list of generic pharmaceuticals and their prices using www.GoodRx.com, based on a convenience sample of the 5 most popular drugs for 10 common medical conditions listed there. Three neuropsychiatric diagnoses were used: Alzheimer's disease, depression and schizophrenia. Seven other diagnoses served as controls: asthma; diabetes mellitus-type II; high cholesterol; hypertension; osteoarthritis; osteoporosis; and urinary tract infection. For each drug, we identified the highest and lowest prices and calculated the mean, median and coefficient of variation (CV). We further calculated the ratios of the highest price to the median price and of the highest to lowest price. We found that the mean price CV was 43%. For neuropsychiatric drugs and controls, it was 61% and 35%, respectively. The mean high-to-median ratio was3.7 for neuropsychiatric drugs and 1.9 for controls. The mean high-to-low ratio was 5.9 for neuropsychiatric drugs and 2.8 for controls. In short, generic medications have high price dispersion, despite public availability of prices. Although our study did not examine why this price dispersion is present, the especially large high-to-low price ratio for neuropsychiatric medications suggests a strategy that exploits vulnerable patients.

## Introduction

A recent news article[1] on the price of aripiprazole (generic *Abilify*) charged by different sellers reported that 30 pills cost between $13.75 and $751.

At first glance, this price disparity makes no sense. Economics’ *Law of One Price* claims that in the absence of trade frictions, and under conditions of free competition and price flexibility, identical goods will sell for the same price. The FDA ensures that generics are identical. Thus, if this law doesn't hold, it must be because one of the conditions is broken.

Of course, idealized conditions are rarely encountered, and thus *some* price dispersion is commonly seen. Scholten and Smith[2], for example, reported that the coefficient of variation (namely, the ratio of the standard deviation to the mean) of consumer good prices ranges from 1.6% to 20.7%. Some buyers might end up paying more than the lowest possible price because of information asymmetry, local monopoly, demand inelasticity or higher search costs, among other reasons[3,4].

But even with those explanations in mind, aripiprazole’s high-to-low price ratio is aptly described as “staggering”[1].

We thus consider two questions. The first is whether the aripiprazole example is anomalous. Although there is a broad literature on price dispersion in other markets, the phenomenon has not been reported with regards to the price dispersion of commonly prescribed generic medications for common medical conditions.

Our second question is whether the price dispersion of generic medications for neuropsychiatric conditions is substantially higher than that of other generic pharmaceuticals. It may be that in the case of mental illness, the very disease that creates the need for the medication might constrain the patient’s ability to comparison shop. If so, greater price dispersion for neuropsychiatric medications might suggest an underlying explanation for the price dispersion of generic drugs in general: namely, a strategy of profiteering that takes advantage of buyers’ constraints.

## Methods

We assembled a list of generic pharmaceuticals and their prices using www.GoodRx.com, in compliance with the site’s Terms and Conditions. GoodRx.com obtains pharmacy prices for prescription drugs by national sampling and offers free public access to this information via a price comparison tool on its website. It has been described as “the superior application for finding the cheapest medication costs.”[5]

Based on a sample size power analysis suggesting a need for approximately 30 drugs, we first selected a convenience sample of 10 common medical conditions from Goodrx.com’s "Health Conditions" page and collected information on 5 generic drugs used for each of the conditions.

Three neuropsychiatric diagnoses were chosen: Alzheimer's disease, depression and schizophrenia. Seven other common medical diagnoses served as controls: asthma; diabetes mellitus-type II; high cholesterol; hypertension; osteoarthritis; osteoporosis; and urinary tract infection.

For each condition, we noted the prices for the five most popular generic pharmaceuticals offered by the eight distinct sellers listed, using the default dosage and volume indicated by GoodRx as most popular. Alzheimer's disease had only 4 generic drugs listed, thus there were only 14 neuropsychiatric medications. Also, two of the medications for osteoarthritis were indicated for another condition as well but counted only once. Thus, there were 33 drugs in the control set and 47 in total. The data were collected in April, 2019.

For each drug, we identified the highest and lowest prices and calculated the mean, median and coefficient of variation.

We further calculated the ratios of the highest price to the median price and of the highest price to lowest price. To minimize the effect of outliers, these ratios were re-calculated for the second highest and lowest prices too. Significant differences in mean values were assessed with Student’s t-test.

## Results

The price dispersion parameters for neuropsychiatric medications and controls are shown in Tables 1 and 2, respectively.

**Table 1:** Price dispersion parameters for neuropsychiatric medications, ordered by high to low price ratios.

Generic Name	Brand Name	Use	High	Low	Mean	Median	Coefficient of Variation	High to median ratio	High to low ratio	2nd highest to median ratio	2nd highest to 2nd lowest ratio
Aripiprazole	Abilify	Schizophrenia	$298.22	$21.52	$110.73	$27.63	110%	10.8	13.9	9.2	11.7
Olanzapine	Zyprexa	Schizophrenia	$97.46	$9.00	$38.84	$15.05	97%	6.5	10.8	6.1	7.0
Risperidone	Risperdal	Schizophrenia	$36.06	$4.00	$16.86	$10.78	77%	3.3	9.0	3.2	4.6
Donepezil	Aricept	Alzheimer's	$55.95	$6.52	$26.06	$12.27	92%	4.6	8.6	4.4	8.3
Quetiapine	Seroquel	Schizophrenia	$72.80	$9.00	$22.63	$12.93	97%	5.6	8.1	2.7	3.0
Escitalopram	Lexapro	Depression	$48.13	$7.35	$21.06	$12.20	85%	3.9	6.5	3.9	6.5
Duloxetine	Cymbalta	Depression	$54.29	$9.00	$26.40	$19.01	56%	2.9	6.0	2.0	2.1
Memantine	Namenda	Alzheimer's	$118.95	$21.96	$55.87	$24.72	82%	4.8	5.4	4.7	5.2
Fluoxetine	Prozac	Depression	$16.59	$4.00	$8.11	$7.23	51%	2.3	4.1	1.4	1.9
Desyrel	Trazodone	Depression	$9.99	$4.00	$7.25	$7.01	23%	1.4	2.5	1.1	1.1
Galantamine ER	Razadyne ER	Alzheimer's	$242.57	$124.04	$182.69	$187.26	20%	1.3	2.0	1.1	1.4
Prochlorperazine	Compazine	Schizophrenia	$17.25	$8.73	$12.95	$12.53	22%	1.4	2.0	1.2	1.5
Sertraline	Zoloft	Depression	$17.10	$8.63	$11.62	$10.47	29%	1.6	2.0	1.4	1.7
Rivastigmine	Exelon	Alzheimer's	$233.90	$125.66	$181.12	$181.65	21%	1.3	1.9	1.2	1.4

**Table 2:** Price dispersion parameters for control medications, ordered by high to low price ratios.

Generic Name	Brand Name	Use	High	Low	Mean	Median	Coefficient of Variation	High to median ratio	High to low ratio	2nd highest to median ratio	2nd highest to 2nd lowest ratio
Pioglitazone	Actos	diabetes	$95.01	$9.00	$41.38	$14.18	100%	6.7	10.6	6.5	8.9
Rosuvastatin	Crestor	high cholesterol	$177.58	$20.25	$65.58	$34.28	98%	5.2	8.8	4.6	7.8
Montelukast	Singulair	asthma	$39.63	$10.05	$20.03	$14.88	60%	2.7	3.9	2.6	3.0
Metformin	Glucophage	diabetes	$15.40	$4.00	$6.54	$5.33	57%	2.9	3.9	1.4	1.8
Losartan	Cozaar	hypertension	$15.00	$4.00	$9.82	$9.59	44%	1.6	3.8	1.5	2.2
Amlodipine	Norvasc	hypertension	$14.76	$4.00	$10.05	$10.08	41%	1.5	3.7	1.4	1.9
Simvastatin	Zocor	high cholesterol	$34.00	$10.00	$19.65	$15.59	53%	2.2	3.4	2.1	3.0
Meloxicam	Mobic	osteoarthritis	$13.51	$4.00	$7.73	$5.65	48%	2.4	3.4	2.2	2.4
Glipizide	Glucotrol	diabetes	$12.13	$4.00	$8.26	$7.89	31%	1.5	3.0	1.4	1.6
Raloxifene	Evista	osteoporosis	$196.76	$70.42	$122.30	$93.31	45%	2.1	2.8	2.1	2.5
Alendronate	Fosamax	osteoporosis	$16.79	$6.36	$10.25	$9.06	42%	1.9	2.6	1.7	2.4
Lovastatin	Mevacor	high cholesterol	$36.63	$13.98	$21.36	$17.64	44%	2.1	2.6	2.0	2.5
Glimepiride	Amaryl	diabetes	$26.01	$10.00	$17.79	$15.92	28%	1.6	2.6	1.5	1.5
Lisinopril	Zestril	hypertension	$9.81	$3.98	$5.66	$5.47	35%	1.8	2.5	1.2	1.7
Estradiol/norethindrone	Activella	osteoporosis	$90.34	$38.86	$67.02	$68.65	37%	1.3	2.3	1.3	2.1
Estradiol	Estrace	osteoporosis	$79.29	$35.06	$56.73	$55.04	39%	1.4	2.3	1.4	2.3
Ibuprofen	n/a	osteoarthritis	$10.05	$4.76	$7.47	$7.24	20%	1.4	2.1	1.2	1.2
Atorvastatin	Lipitor	high cholesterol	$18.90	$9.00	$13.96	$13.77	32%	1.4	2.1	1.3	1.8
Fluticasone/salmeterol	Advair	asthma	$276.16	$132.68	$175.88	$167.68	28%	1.6	2.1	1.2	1.5
Diclofenac	Voltaren	osteoarthritis	$44.64	$21.63	$32.56	$32.38	29%	1.4	2.1	1.3	1.7
Albuterol	Ventolin	asthma	$59.81	$29.63	$49.94	$52.11	19%	1.1	2.0	1.1	1.3
Naproxen	Naprosyn	osteoarthritis	$18.93	$9.58	$12.13	$11.81	25%	1.6	2.0	1.1	1.3
Amoxicillin	Amoxil	urinary tract infection	$9.72	$5.00	$7.70	$8.42	22%	1.2	1.9	1.1	1.6
Glyburide	Micronas	diabetes	$23.21	$12.54	$16.51	$16.47	19%	1.4	1.9	1.1	1.3
Metoprolol-ER	ToprolXL	hypertension	$16.59	$9.09	$13.47	$13.65	20%	1.2	1.8	1.2	1.6
Amoxicillin/clavulanate	Augmentin	urinary tract infection	$26.15	$14.59	$19.95	$19.69	29%	1.3	1.8	1.3	1.7
Cephalexin	Keflex	urinary tract infection	$13.27	$7.46	$11.18	$12.06	19%	1.1	1.8	1.0	1.5
Prednisone	n/a	osteoarthritis	$7.34	$4.14	$5.35	$5.06	19%	1.5	1.8	1.2	1.3
Sulfamethoxazole / trimethoprim	Bactrim	urinary tract infection	$11.05	$6.28	$8.25	$8.77	22%	1.3	1.8	1.1	1.5
Pravastatin	Pravachol	high cholesterol	$43.87	$27.68	$34.71	$32.03	22%	1.4	1.6	1.4	1.6
Doxycycline	Morgidox	urinary tract infection	$20.00	$13.85	$16.78	$16.94	15%	1.2	1.4	1.1	1.4
Hydrochlorothiazide	Microzide	hypertension	$5.68	$4.00	$4.87	$5.04	12%	1.1	1.4	1.0	1.3
Methylprednisolone	medrol	asthma	$18.01	$13.38	$15.60	$16.03	11%	1.1	1.3	1.0	1.2
Pioglitazone	Actos	diabetes	$95.01	$9.00	$41.38	$14.18	100%	6.7	10.6	6.5	8.9
Rosuvastatin	Crestor	high cholesterol	$177.58	$20.25	$65.58	$34.28	98%	5.2	8.8	4.6	7.8
Montelukast	Singulair	asthma	$39.63	$10.05	$20.03	$14.88	60%	2.7	3.9	2.6	3.0
Metformin	Glucophage	diabetes	$15.40	$4.00	$6.54	$5.33	57%	2.9	3.9	1.4	1.8
Losartan	Cozaar	hypertension	$15.00	$4.00	$9.82	$9.59	44%	1.6	3.8	1.5	2.2
Amlodipine	Norvasc	hypertension	$14.76	$4.00	$10.05	$10.08	41%	1.5	3.7	1.4	1.9
Simvastatin	Zocor	high cholesterol	$34.00	$10.00	$19.65	$15.59	53%	2.2	3.4	2.1	3.0
Meloxicam	Mobic	osteoarthritis	$13.51	$4.00	$7.73	$5.65	48%	2.4	3.4	2.2	2.4
Glipizide	Glucotrol	diabetes	$12.13	$4.00	$8.26	$7.89	31%	1.5	3.0	1.4	1.6

The mean price coefficient of variation for all drugs in the sample was 43%. For neuropsychiatric drugs, it was 61% and for controls the coefficient of variation was 35% (Table 3).

**Table 3:** Price dispersion parameters.

Parameter	Neuropsychiatric medications (n = 14)	Controls (n = 33)	P value of comparison (Student’s T-test)
Coefficient of variation	61%	35%	*0*.*0022*
High-to-median ratio	3.7	1.9	*0*.*0018*
Second-highest to median ratio	3.1	1.7	*0*.*0057*
High-to-low ratio	5.9	2.8	*0*.*0005*
Second-highest to second-lowest ratio	4.1	2.2	*0*.*0101*

The mean high-to-median ratio was 2.4 overall. For neuropsychiatric drugs, the mean high-to-median ratio was 3.7. For controls, the ratio was 1.9.

The mean high-to-low ratio for all drugs was 3.7. The mean high-to-low ratio was 5.9 for neuropsychiatric drugs and 2.8 for controls.

## Discussion

In most markets for commodity goods, some price dispersion is routinely encountered. Here, by contrast, we found considerably more than just “some” price dispersion: the CV of drug prices was more than double the upper limit reported for consumer good prices[2]: 43% overall, and 61% for neuropsychiatric drugs.

The high degree of price dispersion seen here for generic medications is noteworthy. First, unlike other consumer goods, in which there may be some perceived, if not real, variation between brands, generic pharmaceuticals are by law completely fungible. Their price dispersion, one might argue, should therefore be considerably lower than that of other consumer goods.

Furthermore, given the complete price transparency offered by sites like GoodRx.com, there should be even less information asymmetry in the generic drug market than seen in other markets and in turn less price dispersion accordingly.

The existence of highly disparate retail prices for generic pharmaceuticals implies that standard price comparison shopping is not happening—even among patients who would be paying out of pocket. Of note, the GoodRx site lists cash prices, not considering health insurance, and thus caters to people who either do not have health insurance or choose not to use it.

This phenomenon of highly disparate retail prices for generic pharmaceuticals further suggests that price transparency might not be as effective for controlling overall health care costs as some hope. It certainly is not working in the market we studied here.

Although possible determinants should be taken into consideration as control variables before drawing any conclusions regarding an explanation of the findings here, the particularly large amount of price dispersion for neuropsychiatric medications might also hint at a rationale for the finding: namely, charging more when the purchasers are limited in their ability to comparison shop. Assuming, not unreasonably, that patients with psychiatric disease are especially limited in their ability to comparison shop, the mean high-to-low price ratio of 5.9 for neuropsychiatric medications, compared to a 2.8 ratio for control-group medications, suggests a strategy that exploits that weakness. Consistent with that, the greatest dispersion is seen for drugs for schizophrenia, which may be associated with greatest limitation on comparison shopping: indeed, patients taking the medications for depression might be more obsessive/detail-oriented than most others; and patients taking medication for Alzheimer's may be so impaired that they have other people, eg caretakers, making the purchase for them.

There are, of course, other plausible explanations for the large amount of price dispersion for neuropsychiatric medications. For example, the duration that the drug has been on the market may be a factor. Aripiprazole, the drug with the highest price dispersion has been sold for approximately 30 fewer years than methylprednisolone, the drug with lowest price dispersion. The presence of alternatives may also affect the amount of price dispersion seen. Goodrx.com, for instance listed 76 drugs in the osteoarthritis category (mean price dispersion in our sample: 0.28) whereas only 39 drugs were listed for schizophrenia (mean price dispersion in our sample: 0.8).

### Limitations

Although the selection of conditions was made before any analysis, their choice was arbitrary, as was the decision to examine exactly five medications per condition. It certainly may be that a different selection of conditions or drugs will yield a different result.

We did not weight our analysis by sales volume. Rather, we used what GoodRx considered to be the most popular drugs in the class and treated them all equally for purposes of the analysis. If those drugs with smaller price dispersion represent a large fraction of total sales for the given condition, our results would be overstated.

In a related point, our measure is based on posted prices, not transactions. If some sellers are not willing to match the discounts offered by others, in practice buyers may respond by shopping elsewhere. Without accounting for this, our measure of price dispersion would be overstated. (Of course, this cuts both ways: sellers advertising very low prices, perhaps even below their wholesale cost, might conveniently find themselves out of stock when a customer appears.)

Our findings also apply only to patients paying for drugs out-of-pocket, and not relying on a pharmaceutical benefit within their health insurance coverage. Cash payers in the United States are a small segment of the market, and thus our findings cannot be extrapolated to represent the market at large. Nonetheless, the demonstration of price dispersion here can serve as an illustrative example of the limited the power of price transparency to control costs.

Last, our conclusions are valid only if GoodRx.com data are accurate. We relied not only on the accuracy of the prices for the given drug, but the identification of the five most popular drugs for the given conditions.

## Conclusions

Generic pharmaceuticals, particularly neuropsychiatric medications, have high price dispersion in the United States. All sellers, presumably, could sell near the market-low price, (buying their inventories from the cheapest retail outlet, if need be). Still, despite the public availability of prices, many choose not to. Thus, transparency alone may not be enough to guarantee fair and efficient pricing and complete access to care.

## Supporting information

S1 File The prices of all drugs, by pharmacy. (XLSX)
